# Change in self-construal: a repertory grid technique study of women admitted to a Mother and Baby Unit

**DOI:** 10.3389/fpsyt.2024.1424010

**Published:** 2024-11-15

**Authors:** Eleanor E. Wozniak, Dougal Julian Hare, Lynsey Gregg, Anja Wittkowski

**Affiliations:** ^1^ Division of Psychology and Mental Health, School of Health Sciences, The University of Manchester, Manchester, United Kingdom; ^2^ The Perinatal Mental Health and Parenting (PRIME) Research Unit, Greater Manchester Mental Health National Health Service (NHS) Foundation Trust, Manchester, United Kingdom; ^3^ Manchester Academic Health Science Centre, The University of Manchester, Manchester, United Kingdom

**Keywords:** maternal, intervention, perinatal, mental health, inpatient care, *Personal Construct Theory*, beliefs, attitudes

## Abstract

**Introduction:**

Pregnancy and the postnatal period represent a time of heightened risk for women to experience mental health difficulties. Some mothers may require specialist inpatient psychiatric support made available through Mother and Baby units (MBUs). Although there is evidence of the therapeutic benefits of MBUs, many studies have utilised methodologies vulnerable to interviewer and social desirability biases. The repertory grid technique (RGT), derived from personal construct theory (PCT), has been successfully used to explore how the way in which a person thinks about and defines the self (i.e., self-construal) changes following therapeutic intervention in samples of people experiencing mental health conditions. Therefore, this study aimed to explore change in maternal self-construal following MBU admission, utilising the RGT, thereby enhancing our understanding for the therapeutic role of MBU admissions in women’s mental health recoveries.

**Methods:**

Participants were recruited from two MBUs in England. RGT was undertaken with participants shortly after admission and again at discharge, allowing for comparisons between grids to assess change in how a mother viewed herself in relation to certain aspects of the self (e.g., *ideal self*) and other people, a concept referred to as *construing* in PCT. Data were analysed using principal component analysis, Slater analysis, and content analysis.

**Results:**

There were 12 participants who completed repertory grids at admission, with eight (66.67%) participants also completing discharge grids. Most of the eight participants demonstrated improvements in overall self-esteem and self-esteem as a mother, a shift towards a more positive self-perception, and increased construed similarity between the self and positively construed others, and construing became more varied. Conversely, a few participants displayed a reduction in self-esteem, particularly in the maternal role and increased construed similarity between the self and negatively construed others, and construing became more rigid.

**Conclusions:**

All participants exhibited changes to construing during their MBU admission, with most participants displaying positive changes to self-esteem and self-perception and a more adaptive process of construing. Potential implications are offered for service users, families, clinicians, and stakeholders. Recommendations for future research are also provided.

## 1 Introduction

The transition to motherhood, starting in pregnancy, giving birth, and the 12-month postnatal period are significant life events for women (1, 2), a time defined as the perinatal period (3). This transition to motherhood, a catalyst for significant shifts in a woman’s identity (4), can challenge a woman’s sense of self (5) and encompass shifts in self-concept which negatively impact mental health (6).

Women often have established personal standards that they aspire to adhere to as a mother, which can be referred to as personalised ideals (7). Women can also be exposed to societal myths surrounding motherhood (8) and societal principles conceptualising what constitutes a ‘good mother’ (9, 10), which can contribute to forming a woman’s internalised societal motherhood ideals. One example is the ‘Good Mother’ ideology (10). It is the integration of these personalised and internalised societal ideals and the woman’s actual experiences of being a mother that happens during the formation of the ‘motherhood identity’ (4, 11, 12).

However, an incongruity can occur between the woman’s actual experience and their *pre-existing* ideals of motherhood (5). Women who persistently experience greater discrepancy between their prior ideals and lived reality are at a greater risk of experiencing emotional difficulties, such as anxiety (13), low self-esteem (13), severe postpartum depression (14, 15), feelings of inadequacy (16), or shame and guilt (7, 17). Perceiving oneself as an ineffective or non-nurturing mother can contribute to difficulties, such as the development of postnatal depression (18). It is not only the comparison of their actual experience to internalised ideals that appears to influence the emotional well-being of the mother, because the process of external comparison with other mothers is also important: higher rates of depression are observed in mothers who negatively compare their actual motherhood experience to that of other mothers (19). Conversely, as mothers who possess positive self-esteem and a healthy self-concept tend to experience higher emotional well-being (20, 21), decreasing the discrepancies between internalised motherhood ideals and a person’s lived reality can play a vital role in achieving a successful transition to motherhood (22).

The perinatal period, especially the first 30 days postpartum, represents one of the highest-risk periods for women in their lives for the development of new and/or recurrence of pre-existing mental health conditions (23–27). Mental health conditions, such as depressive disorders, anxiety disorders, and psychosis, are the most common difficulties to occur in the perinatal period (24, 28). Globally, approximately one in five women will experience a mental health condition in the perinatal period, with disparities observed across low-, middle-, and high-income countries (29). The rates of perinatal mental health conditions in the UK are reflective of the global average, with approximately 20% of British women experiencing such difficulties (30).

In the UK, approximately 4 in 1,000 women with perinatal onset mental health conditions require more intensive psychiatric support accessed through an inpatient admission, ideally to a specialist inpatient Mother and Baby Unit (MBU) (30, 31). MBUs offer joint mother–infant inpatient admissions, facilitating psychiatric, psychological, and psychosocial assessment and intervention for the mother while also enriching the mother–infant attachment (30–32). Effective care and treatment of a mother’s mental health during the perinatal period is paramount because untreated perinatal mental health conditions can result in adverse effects for both the mother and her developing child (33, 34). In their systematic review of published psychological guidance for perinatal mental health, O’Brien et al. (35) emphasised the importance of targeting both maternal mental health difficulties and the mother–infant dyad to achieve effective outcomes, a goal made possible through a joint MBU admission.

The benefits of MBUs have been noted. In their systematic review of 23 studies, Gillham and Wittkowski (36) identified that MBU admission improved maternal clinical symptoms, maternal confidence, child development, and the mother–baby relationship, including enhanced infant attachment. The finding of improved maternal outcomes following MBU admission was upheld by a more recent systematic review of 44 studies by Connellan et al. (37) and by the empirical studies of Branjerdporn et al. (38), Stephenson et al. (39), and Wright et al. (40).

MBU co-admission is preferable in supporting maternal mental health conditions during the perinatal period in many ways over mother-only general psychiatric hospital admission (41), and women admitted to an MBU seem to express high levels of care satisfaction (38, 42). The *Effectiveness of Services for Mothers with Mental Illness* (ESMI) research programme report (43) noted that mother–infant separation resulting from individual maternal hospitalisation can be perceived as traumatic by mothers and detrimental to the mother–infant dyad, ultimately hindering recovery. Indeed, mothers expressed that being with their baby had been an important factor in recovery (41, 44). However, most studies illustrating the benefits of MBU admission for both mother and baby utilised methodological approaches susceptible to social desirability and researcher biases, such as interviews, observational methods, standardised psychometrics, and rating scales (45).

A more appropriate methodology is the repertory grid technique (RGT) (46), an assessment tool derived from *Personal Construct Theory* (PCT0) (47). PCT postulates that people engage in an ongoing process of utilising an idiosyncratic system of *personal constructs* to understand and distinguish between *elements* within themselves and among others in the world, a process termed *construing* (48). Examples of elements include the *actual self*, *ideal self*, and *person of trust* (49). The *constructs* that a person develops are bipolar in nature, for instance, ‘undeserving–deserving’ (p. 176) (50) and shape expectations and interpretation of events while being continually open to revision in response to experience (47). This process of *construing* itself falls on a ‘loose–tight’ continuum with extremes of very loose or very tight construing, constituting a threat to psychological well-being. ‘Loose’ construing occurs when an *element* is positioned on opposing ends of *construct* poles on different occasions and results in variable predictions and a disorganised view of the self and world (47). Conversely, excessively ‘tight’ construing results in consistent and precise but unvarying predictions with overly ‘tight’ construing resulting in ‘all or nothing’ thinking (51).

An advantage of RGT is that via elicitation of idiosyncratic definitions of *elements* and *constructs*, it can reveal implicit attitudes that may not be shared in interviews (52). Similarly, it can be used to detect clinically meaningful changes in construing following therapeutic interventions that cannot be identified by standard questionnaires (53). Overall, RGT enables a person’s subjective experience to be subjected to objective analysis, thereby reducing researcher bias (54).

As such, RGT has been effectively utilised in mental health research, particularly inpatient samples, including those experiencing psychosis, anxiety, and depression (49, 55–58), to examine changes in construing of people experiencing a mental health condition following therapeutic intervention (59–61). A key aspect of RGT in such studies is the facility to conduct idiosyncratic assessment across different time points with very small sample sizes of up to eight participants (59, 62, 63). Furthermore, Wittkowski et al. (64) used RGT to examine the experiences of compassionate care during an MBU admission.

No study to date has investigated the construals and construing in mothers at admission to an MBU and assessed for changes in construing following an MBU admission, to explore the clinical benefits of MBU care in supporting maternal mental health. Using the RGT, the preliminary aim of this current study was to assess how mothers who experienced an acute mental health crisis that necessitated MBU care construed themselves at admission. Furthermore, this exploratory study sought to answer the following question: “How does a mother’s self-concept, captured through the way she construes herself, particularly how she construes herself as a mother, change during an admission to an MBU?”.

## 2 Materials and methods

### 2.1 Design

The study utilised a within-subject repeated measures design, with participants acting as their own control. It used RGT (46) to investigate maternal construal and process of construing at admission, and then assess for change to these areas during an MBU admission.

### 2.2 Ethical and other approvals

Relevant approvals were obtained from the NHS Research Ethics Committee (REC) and Health Research Authority (HRA) (reference: 23/WA/0020), and local NHS Trusts Research and Innovation departments (reference: x648). In line with UK legislative frameworks for best practice, experts by experience were consulted on the research design process (65). We liaised with at least one member of the University’s *Community Liaison Group* (its members include services users, people with lived experiences, and carers) in the development stage of this study, who recognised the potential benefits of this study and advised on some procedural aspects.

### 2.3 Participant inclusion and exclusion criteria

Participants, considered eligible for inclusion, were 1) aged 18 years or over, 2) admitted to a participating MBU within the last 4 weeks, 3) presenting with any mental health diagnosis, 4) pregnant or had delivered a baby in the last 12 months, 5) proficient in the English language, and 6) able to give informed consent. RGT methodology is not language-specific, but given limited resources to facilitate appropriate translation services, participants not fluent in English were excluded.

### 2.4 Recruitment and setting

Participants were recruited from two MBUs across two NHS Trusts in the Northwest of England between March 2023 and February 2024. The 8–10-bed MBUs provided similar specialist care, and the staff teams on both units comprised psychiatric and medical staff, psychiatric nurses, nursery nurses, clinical psychologists, and occupational therapists.

Participants were recruited via two main methods. Firstly, interested participants were able to contact the main researcher directly following study advisements to register an interest in taking part. On contact, a participant information sheet was shared. Alternatively, ward-based clinical staff identified potential participants and asked them to complete a consent to contact form if they were interested in participating. On receipt of the consent to contact form or self-directed contact, the main researcher then contacted the potential participant and ascertained eligibility, and the first assessment session was arranged.

### 2.5 Questionnaire data collection

A demographic questionnaire, specifically designed for this study, was used alongside the *Clinical Outcomes in Routine Evaluation - Outcome Measure* (CORE-OM) (66) to contextualise individual experiences and the sample of participants. The CORE-OM is a 34-item self-report measure which assesses a person’s current psychological global distress, and the efficacy and effectiveness of psychological intervention (67). Responses on the scale range from 0 (not at all) to 4 (most or all the time), yielding possible scores between 0 and 136. For a total score of current psychological global distress, a score of 1–20 is deemed a ‘healthy’ level of distress and a score of 85+ indicates a ‘severe’ level of distress. The CORE-OM was scored in line with standardised scoring procedures (68) and was based on the *mean rating applied to each sub-scale*, with a score of 1.00 or above indicative of exceeding the clinical cut-off. The CORE-OM is recommended for UK perinatal mental health services (69) and has good internal reliability, test–retest reliability, convergent validity with other measures, and sensitivity to change (67). Furthermore, the CORE-OM can successfully assess change in clinical recovery (70).

### 2.6 Repertory grid procedure

To develop the repertory grid, participants were presented with the following 10 elements: 1) *actual (current) self*, 2) *ideal self*, 3) *actual (current) self as mother*, 4) *ideal self as mother*, 5) *ideal other mother*, 6) *other mother on MBU*, 7) *friend I know who is a mother*, 8) *self before becoming a mother*, 9) *ideal future self*, and 10) *ideal future self as a mother*. These elements were based on the repertory grid literature (46, 52) and their relevance to the perinatal field and were predetermined by the authors, who have expertise in utilising repertory grid methodology and working clinically in mental health. The triadic difference method was used to generate bipolar constructs (54, 71) and involved presenting the participant with three randomly selected elements at one time and asking them to consider “*How are two of these similar to one another (emergent construct) and different from the third (implicit construct)?*”. Participants were then asked to describe in detail and give behavioural examples of each construct pole that had been developed to ensure the specific meaning was identified. This process was repeated to ensure each element was used to generate constructs at least once, and until the participant was unable to generate any more constructs. The participant was then asked to select the construct end which was preferred and to rank each element along the elicited construct poles, using a 10-point scale, to generate the repertory grid. The ranking approach was chosen over alternative methods, such as rating elements on a Likert scale, to ease the procedural load on participants who were presenting with significant mental health difficulties. Elements used to elicit the construct were rated first and the remaining elements randomly thereafter. To maintain anonymity, the elements were identified by initials. The same elements were used to allow greater comparability across multiple-participant grids. On repetition of the repertory grid at the second assessment session, the same elements and same constructs developed and utilised at the first assessment session were used, which enabled comparison of a participant’s repertory grids across time, to explore for commonalities and differences.

### 2.7 Procedure

The main researcher (EW), who was not an MBU staff member and hence conducted the study independently, completed two assessment sessions with participants at two time points to enable construing to be captured at admission and then discharge, with the intention of attributing change in construing specifically to the MBU admission. In the current study, the therapeutic intervention was defined as the admission to an MBU. The first assessment session took place within the first 4 weeks of admission, allowing participants time to settle on the ward and for acuity of difficulties to lessen. The second assessment session took place at discharge or up to 3 months post discharge. The procedure for assessment sessions one and two were identical, and the same repertory grid was used at each assessment session. The assessment sessions used a one-to-one format, each lasting 45 min–120 min. Assessment sessions were arranged for a convenient time for the participant. After providing informed consent, participants were asked to firstly complete the demographic questionnaire, followed by the CORE-OM (66), and then time was spent developing and completing the repertory grid. Following each assessment session, participants were provided with a debrief form and an e-voucher worth £10 each to thank them for their time and participation.

### 2.8 Data analysis

Demographic data were analysed using the Statistical Package for Social Sciences v29.0 (72). CORE-OM scores were produced at admission and discharge, and the numerical difference in the CORE-OM scores between administrations was calculated. A Reliable Change Index (RCI) was used to assess for reliable change in the level of psychological global distress over time. For the degree of change to be classified as reliable, the change in the *mean rating applied to sub-scales* must have been greater than 0.5 (73). Demographic data and CORE-OM scores were then tabulated.

Individual repertory grids were analysed using the repertory grid analysis package Idiogrid version 2.4 (74). Principal component analysis (PCA) (75) was then performed on each participant’s admission repertory grids and, if available, discharge repertory grids, to explore construing at each time point (52). The process of construing was established using the eigenvalue, i.e., the percent variance accounted for by the first principal component, with eigenvalues categorised as ‘tight’ or ‘loose’, with a greater eigenvalue indicative of ‘tighter’ construing (51). Observations were made between eigenvalues obtained from admission and discharge grids, to assess construing at admission, and the evidence of any change in construing over time. Changes in eigenvalues were subjected to further analysis (i.e., Wilcoxon signed rank test) to assess the significance of any changes observed.

To explore construal at admission and discharge, Slater analysis (76) (Slater, 1967) was used to establish the mean Euclidean distance between pairs of elements of interest from all repertory grids. The use of the same elements and constructs during both repertory grid administrations enabled differences in Euclidean distance between elements to be measured across time. Elements deemed to be construed as very similar were those in which the inter-element Euclidean distance was less than 0.5, whereas those deemed as highly dissimilar had a distance greater than 1.50 (51). The Euclidean distance between the following pairs of elements were calculated: a) *actual (current) self as mother* and *ideal self as mother*, to establish a measure of self-esteem as a mother; b) *actual (current) self* and *ideal self*, to establish a measure of self-esteem; c) *actual (current) self as mother* and *non-self* elements, to assess the construal of non-self elements; and d) *self before becoming a mother*, *ideal self*, and *actual (current) self*, to explore the construal of self before becoming a mother.

To ascertain construal at admission and assess for any change over time, yielded by the PCA (75), the mean ranked position of specific elements on each elicited construct pole and elements’ mean ranked position comparative with other elements were calculated on all repertory grids. The higher the mean ranked position, the more closely the negative pole of the construct (non-preferred) applied to the element. This analysis facilitated exploration of self-perception, by studying whether the *actual (current) self* and the *actual (current) self as mother* tended to be construed more positively or negatively. In addition, construal of *non-self* elements, by investigating how the *actual (current) self as mother*, *other mother on MBU* and *friend I know who is a mother* were positioned relative to one another along construct dimensions.

Change in construing over the period of MBU admission was calculated by establishing changes in the Euclidean distance between the pairs of elements of interest and changes to the mean ranked position of elements along elicited construct poles between the admission and discharge repertory grids of each participant. Changes in Euclidean distances and mean ranked position of elements along elicited construct poles were subjected to further analysis (i.e., Wilcoxon signed rank test) to assess the significance of any changes observed.

Finally, content analysis was conducted across all repertory grids, using the Classification System for Personal Constructs (CSPC) (77) to explore commonality in the elicited constructs. This analysis involved categorising all constructs elicited by participants into one of eight areas, six comprehensive areas, and two supplementary areas (77): 1) *Moral*, 2) *Emotional*, 3) *Relational*, 4) *Personal*, 5) *Intellectual/Operational*, 6) *Values/Interests*, 7) *Existential* (supplementary area), and 8) *Concrete* (supplementary area).

## 3 Results

### 3.1 Participant characteristics

**Figure 1:**
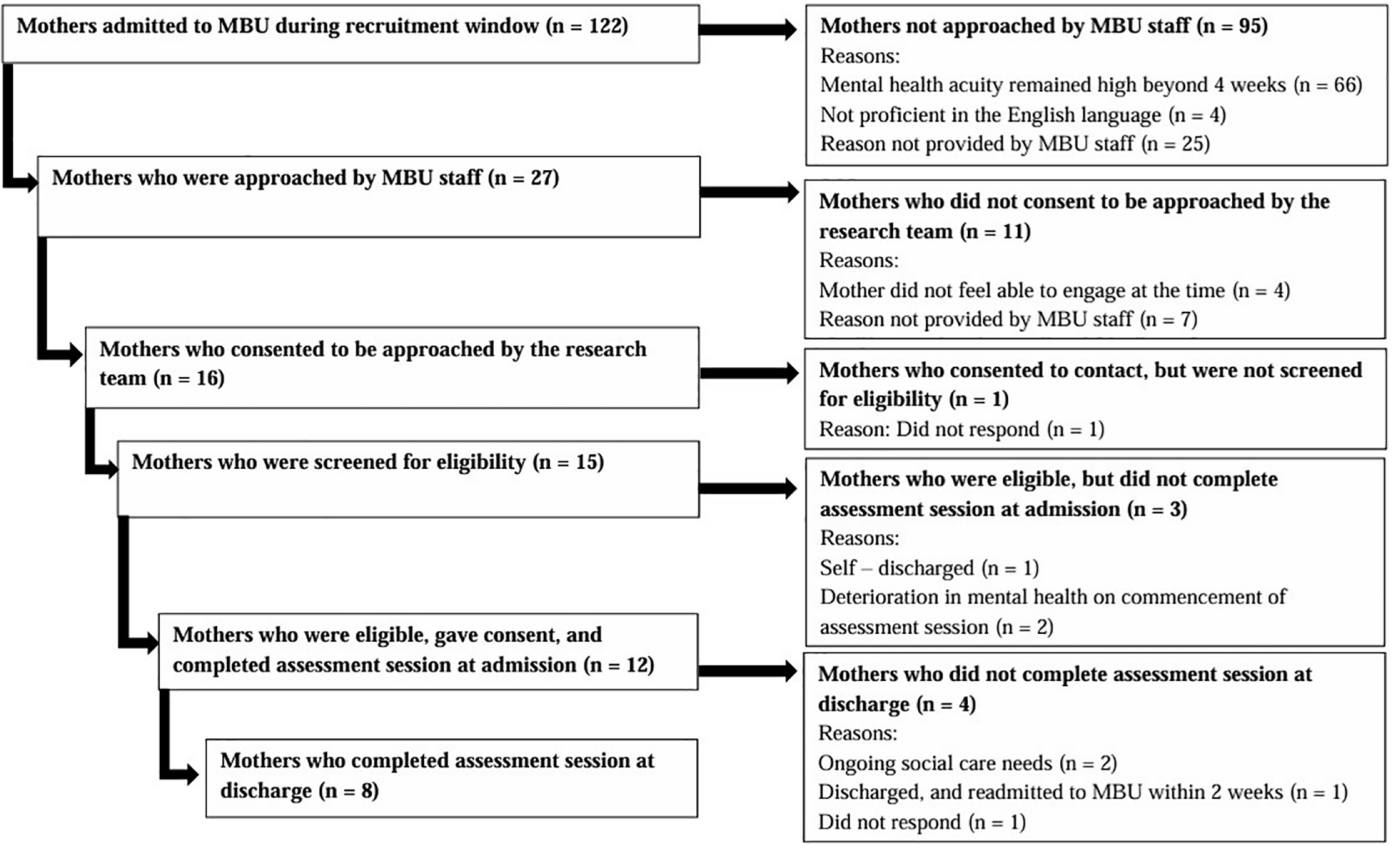
Consort diagram illustrating flow of MBU participants through the study.

**Figure 1** illustrates the number of mothers admitted to the MBUs during study duration and the flow of participants through the study. Eight participants completed the RGT assessment session at admission and discharge. Four participants undertook the admission RGT assessment session only for various reasons including ongoing social care needs that affected their engagement (*n* = 2), readmission to an MBU within 2 weeks of discharge rendering them ineligible (*n* = 1), and not responding to contact from the research team (*n* = 1). There were 11 of the 12 participants who were admitted to one of the two MBUs utilised for recruitment, and all participants who completed RGT assessment sessions at discharge were admitted to the same MBU. All participants were recruited by one recruitment method and were identified by MBU staff as potential participants (*n* = 12). All admission RGT assessment sessions took place on the MBU (*n* = 12), whereas discharge assessment sessions were conducted either on the MBU (*n* = 4), or in person, at the participant’s home (*n* = 4), depending upon participant preference.

The demographic characteristics of all 12 participants are displayed in **Table 1**. Participants were aged 21 to 43 years (*M* = 30.92, SD = 6.13), and almost all described their ethnic origin as White British (*n* = 11). Most participants were either married (*n* = 4) or in a relationship with the father of their child (*n* = 6). Most participants were also employed full-time (*n* = 11), and all employed participants were on maternity leave at the time of participating in the study. Education was achieved to GCSE (*n* = 1), A-Level/BTEC (*n* = 6), and university degree (*n* = 5).

**Table 1:** The demographic characteristics of all 12 participants.

Demographic characteristic	*n* (%)
Age (years)	
Mean (SD)	30.92 (6.13)
Range	21–43 years
Ethnicity	
White British	11 (91.67%)
Black	0 (0.00%)
Asian	1 (8.33%)
Current relationship status	
Single	2 (16.67%)
Married	4 (33.33%)
In a relationship and/or living together	6 (50.00%)
Highest level of education	
GCSEs, CSEs, or O-levels	1 (8.33%)
A levels/BTEC	6 (50.00%)
University degree	5 (41.67%)
Occupation	
Employed: full time	11 (91.67%)
Unemployed	1 (8.33%)
Previous mental health difficulties	
Yes	10 (83.33%)
No	2 (16.67%)
Primary mental health diagnoses	
Anxiety disorders (e.g., perinatal anxiety)	3 (25.00%)
Trauma-related disorders (e.g., PTSD)	2 (16.67%)
Depressive disorders (e.g., postnatal depression)	5 (41.67%)
Psychotic and related disorders (e.g., postpartum psychosis)	2 (16.67%)
Current admission status	
Informal	10 (83.33%)
Section 2 or 3 of the MHA	2 (16.67%)
Number of other inpatient admissions	
0	8 (66.67%)
1–3	2 (16.67%)
3+	2 (16.67%)
Number of children	
1	4 (33.33%)
2–3	8 (66.67%)
Number of pregnancies	
1	3 (25.00%)
2–3	7 (58.33%)
3+	2 (16.67%)
Length of MBU admission (days)	
Mean (SD)	70.25 (29.19)
Range	41–122

SD, standard deviation; *n*, number of participants; MHA, Mental Health Act (UK Government, 1983); MBU, mother and baby unit; PTSD, posttraumatic stress disorder.

Prior to the current episode of mental ill health, most participants expressed experiencing previous mental health difficulties (*n* = 10). Although for most participants, this was their first hospital admission (*n* = 8). Participants were often diagnosed with more than one condition; however, primary diagnoses included depressive disorders (*n* = 5), anxiety disorders (*n* = 3), trauma-related disorders (*n* = 2), and psychotic-related disorders (*n* = 2). The duration of MBU admission for participants ranged between 41 and 122 days (*M* = 70.25, SD = 29.19). The majority of participants were informally admitted to the MBU (*n* = 10), and only two participants were admitted under a section of the Mental Health Act (78) (MHA; UK Government, 1983).

### 3.2 Psychological distress from questionnaire data

At admission, the overall *mean rating applied to all sub-scales* for the sample of 12 participants was above the clinical cut-off (*M* = 2.22, SD = 0.85; see **Table 2**); however, there was a range in the *mean rating applied to all sub-scales* between 0.47 and 2.97. At discharge, the overall *mean rating applied to all sub-scales* remained above the clinical cut-off for the sample of eight participants (*M* = 1.30, SD = 0.63), and the *mean rating applied to all sub-scales* ranged between 0.35 and 2.26. In accordance with the RCI, there was a reliable decrease in the *mean rating applied to all sub-scales* between admission and discharge.

**Table 2:** Clinical outcome data for admission and discharge.

Total *n*	Admission CORE-OM assessment				
	All	Well-being	Problems	Functioning	Risk
Mean total raw score (SD)	75.50(29.03)	12.42 (4.72)	33.08 (10.24)	23.83 (11.11)	6.17 (5.41)
Range in total raw score	16-115	2-16	10-48	4-37	0-14
Mean rating applied (SD)	2.22 (0.85)	3.10 (1.18)	2.76 (0.85)	1.99 (0.93)	1.03 (0.90)
Range in mean rating applied	0.47-2.97	0.50-4.00	0.83-4.00	0.33-3.08	0.00-2.33
	Discharge CORE-OM assessment				
	All	Well-being	Problems	Functioning	Risk
Mean total raw score (SD)	43.00 (21.68)	7.50 (3.34)	19.75 (10.17)	13.75 (7.89)	2.00 (2.73)
Range in total raw score	12-77	1-12	7-34	3-24	0-7
Mean rating applied (SD)	1.30 (0.63)	1.88 (0.83)	1.65 (0.85)	1.15 (0.66)	0.33 (0.45)
Range in mean rating applied	0.35-2.26	0.25-3.00	0.58-2.83	0.25-2.00	0.00-1.17
Reliable change	Yes: decrease	–	–	–	–

*This table is a reduced version and is presenting the CORE-OM data of the sample of 12 participants at admission and sample of 8 participants at discharge collectively. Individual data is not presented to protect the anonymity of participants.

SD, standard deviation; n, sample; -, not applicable; CORE-OM, Clinical Outcomes in Routine Evaluation - Outcome Measure (Evans et al., 2000); reliable change, a reliable change in the mean rating applied across all sub-scales, with an increase or decrease of >0.5 indicating reliable change.

Of the eight participants who completed admission and discharge grids, seven demonstrated a decrease greater than 0.5 in the *mean rating applied to all sub-scales*, indicating that these participants exhibited reduced levels of global psychological distress over the course of their MBU admission. Three of these seven participants showed a *mean rating applied to all sub-scales* at discharge, which was below the clinical cut-off for heightened psychological distress. One participant showed an increase in this score; however, this was a non-reliable change (i.e., <0.5 increase in *mean rating applied to all sub-scales*).

### 3.3 Summary of repertory grids

#### 3.3.1 Self-esteem, self-perception, and construing of non-self elements at admission

Participants tended to experience low self-esteem at admission to the MBU, indicated by a high level of dissimilarity between the *actual current self* and the *ideal* self (sample *M* Euclidean distance = 1.36; see **Table 3**); this pattern of construing was observed for all 12 participants. Similarly, participants presented with low self-esteem as a mother at admission, illustrated by a high level of dissimilarity between the *actual current self as mother* and the *ideal self as mother* (sample *M* Euclidean distance = 1.32). No participant construed the *actual current self as mother* and the *ideal self as mother* as highly similar at admission (i.e., a Euclidean distance of <0.5 between these two elements). Participants tended to construe the *actual current self* (sample *M* ranked position along construct poles = 8.71) and the *actual current self as mother* (sample *M* ranked position along construct poles = 8.45) extremely negatively. These findings implied that they held a highly negative self-perception for both their holistic self and their self as a mother at admission.

**Table 3:** Euclidean distances (standardised) and mean ranked ratings used to calculate self-esteem, self-perception, and construal of non-self elements at admission.

*p*	Self-esteem				Self-perception			Construal of non-self elements			
	ED: actual self as mother and ideal self as mother	ED: actual self and ideal self	ED: self before becoming a mother and ideal self	ED: self before becoming a mother and actual self	Mean ranked position of actual self	Mean ranked position of actual self as mother	Mean ranked position of self before becoming a mother	ED: actual self as mother and other mother on MBU	ED: actual self as mother and friend who is a mother	Mean ranked position of other mother on MBU	Mean ranked position of friend who is a mother
**1**	1.80	1.23	0.97	0.27	9.00	10.00	7.88	0.71	1.03	7.00	5.63
**2**	0.99	0.70	0.99	0.64	7.80	6.50	9.60	0.54	0.42	7.70	6.80
**3**	1.05	1.70	1.07	1.22	8.27	8.18	4.73	0.47	0.78	9.45	5.64
**4**	0.91	1.25	0.75	1.14	6.69	8.38	3.15	0.84	1.46	7.69	3.62
**5**	1.42	1.31	0.71	0.77	9.57	9.00	6.79	0.47	0.55	7.71	6.93
**6**	0.90	1.53	0.79	0.79	7.90	6.40	4.80	0.75	0.69	8.30	7.20
**7**	1.62	1.53	1.21	0.39	8.92	8.67	7.67	1.21	1.24	5.67	5.42
**8**	1.38	1.00	0.59	0.63	7.80	8.00	6.00	0.57	0.82	8.80	6.40
**9**	1.69	1.56	1.24	0.93	9.83	8.42	7.33	0.37	0.56	7.42	6.17
**10**	1.49	1.98	0.88	1.22	9.56	9.44	4.56	0.36	0.58	8.00	7.00
**11**	1.36	1.28	0.65	1.43	9.40	9.40	3.80	0.41	0.85	8.10	6.00
**12**	1.17	1.19	0.52	0.85	9.80	9.00	6.50	0.39	1.30	7.70	4.40
**Sample *M***	**1.32**	**1.36**	**0.86**	**0.86**	**8.71**	**8.45**	**6.07**	**0.59**	**0.86**	**7.80**	**5.93**

ED, Euclidean distance; *M*, mean; *p*, participants; MBU, mother and baby unit.

Mean ranked rating score ranged from 1 to 10, with 1 = the closest positive pole of the construct (preferred) is applied to the element and 10 = the closest negative pole of the construct (non-preferred) is applied to the element. Participants shaded in grey did not complete the discharge repertory grid.

Participants tended to construe greater similarity between the *actual current self as mother* and the *other mother on the MBU* (sample *M* Euclidean distance = 0.59), relative to the *actual current self as mother* and a *friend who is a mother* (sample *M* Euclidean distance = 0.86) at admission, with the exemption of two participants (*p* numbers are withheld throughout to protect participant anonymity) who did not demonstrate this pattern of construing. These findings suggested that, in general, participants tended to construe themselves as more similar to another mother with a mental health condition that necessitated an inpatient admission, compared with a friend who is a mother, residing in the community. All participants construed a *friend who is a mother* more positively (sample *M* ranked position along construct poles = 5.93) than the *other mother on the MBU* (sample *M* ranked position along construct poles = 7.80). All 12 participants construed a *friend who is a mother* more positively than the *actual current self* (sample *M* ranked position along construct poles = 8.71), and 10 participants also construed this *non-self* element more positively than the *actual current self as mother* (sample *M* ranked position along construct poles = 8.45). The same two participants mentioned above demonstrated an opposite pattern of construing to most participants, construing the *actual current self as mother* more positively than a *friend who is a mother.* Participants construed the *other mother on the MBU* more positively than the *actual current self*, with the exception of four participants. Similarly, participants construed the *other mother on the MBU* more positively than the *actual current self as mother*; however, three of the same participants, alongside another participant, did not demonstrate this pattern of construing.

*Self before becoming a mother* (sample *M* ranked position along construct poles = 6.07) was construed more positively than both the *actual current self* and the *actual current self as mother* by all but one participant, suggesting that participants generally held a positive perception of their past self. Participants construed the *self before becoming a mother* more positively than the *other mother on the MBU*, except for three participants. Despite the sample mean ranked position along construct poles being greater for the *self before becoming a mother* (sample *M* = 6.07) than a *friend who is a mother* (sample *M* = 5.93), seven participants construed the *self before becoming a mother* more positively compared with a *friend who is a mother.* Therefore, the current findings indicated that participants admitted to an MBU tended to construe the *self before becoming a mother* more positively than both *non-self* elements. Interestingly, the *self before becoming a mother* was construed as equally dissimilar to the *ideal self* (sample *M* Euclidean distance = 0.86) and the *actual self* (sample *M* Euclidean distance = 0.86).

Overall, participants experienced low self-esteem in general and low self-esteem as a mother, in conjunction with a negative self-perception, at admission to an MBU. In addition, participants with a low self-esteem and a negative self-perception at admission also perceived their past self positively. They construed mothers who were not accessing support from an MBU for their mental health needs more positively compared with mothers who required psychiatric inpatient admission. Then, respectively, participants generally construed other mothers accessing MBU support more positively, compared with themselves; however, this was not a pervasive finding throughout the sample of 12 participants. Together, these findings suggested that participants typically construed other mothers in general, irrespective of whether they were admitted to an MBU or not, more positively, compared with how they construed themselves. Moreover, participants tended to construe themselves as more similar to the other mothers on the MBU compared with a friend, inferring that participants generally construed greater similarity between themselves and the non-preferred *non-self* element. In the instances where participants construed the other mothers on the MBU more negatively relative to themselves, some of these participants did demonstrate the pattern of construing whereby the *actual current self as mother* was construed as more similar to the *non-self* element that was perceived negatively (i.e., *other mother on the MBU*). Therefore, despite not illustrating the common pattern of construing, i.e., the *other mother on the MBU* being perceived more positively relative to the self, the pattern of construing that was exhibited substantiated the notion that participants tended to hold a negative self-perception at admission.

Some specific observations were noted. One participant did not demonstrate some of the typical patterns of construing observed in the sample. Firstly, she held a more positive self-perception for her current self as a mother, as demonstrated by more positive construing of the *actual current self as mother*, compared with both *non-self* elements. She also construed the *actual current self as mother* as more similar to a *friend who is a mother*, the *non-self* element she construed more positively, compared with the *other mother on the MBU*. To offer more understanding for this pattern of construing, contextual information showed that this mother had been engaging in intensive psychological therapy prior to admission to the MBU, which she reflected had provided several therapeutic benefits. She shared her appreciation for her ‘internal strength’ and reflected on a sense of ‘personal pride,’ in relation to managing internal distress. This contextualisation lends support to the previous inference that construing the self negatively tended to associate with construing the self as more similar to the *other mother on the MBU*, who was construed negatively.

#### 3.3.2 Self-esteem, self-perception, and construing of non-self elements at discharge and change during MBU admission

Of the 12 participants who completed admission repertory grids, eight participants completed discharge repertory grids (see **Figure 1**). Discharge repertory grids were subjected to Slater analysis, to calculate construal at discharge (see **Table 4**). Comparisons between admission and discharge grids were then made and numerical values extracted from the repertory grids examined using the Wilcoxon signed-rank test to explore any changes in construal during MBU admission (see **Table 5**).

**Table 4:** Euclidean distances (standardised) and mean ranked ratings used to calculate self-esteem, self-perception, and construal of non-self elements at discharge.

*p*	Self-esteem				Self-perception			Construal of non-self elements			
	ED: actual self as mother and ideal self as mother	ED: actual self and ideal self	ED: self before becoming a mother and ideal self	ED: self before becoming a mother and actual self	Mean ranked position of actual self	Mean ranked position of actual self as mother	Mean ranked position of self before becoming a mother	ED: actual self as mother and other mother on MBU	ED: actual self as mother and friend who is a mother	Mean ranked position of other mother on MBU	Mean ranked position of friend who is a mother
**1**	–	–	–	–	–	–	–	–	–	–	–
**2**	–	–	–	–	–	–	–	–	–	–	–
**3**	0.54	0.53	0.96	1.31	4.09	3.27	6.91	1.63	0.94	9.91	7.09
**4**	1.17	1.13	0.89	0.65	6.15	6.92	5.23	0.89	0.92	6.62	6.69
**5**	1.20	0.85	0.76	0.53	8.43	7.14	7.50	0.70	0.45	9.14	7.71
**6**	1.20	1.59	0.85	1.68	7.20	6.70	4.20	0.95	0.95	7.60	7.40
**7**	1.35	1.07	0.89	0.31	8.50	8.67	8.08	0.72	0.86	6.92	6.42
**8**	1.04	0.83	0.75	1.05	7.10	6.30	4.40	0.94	0.72	9.80	6.90
**9**	–	–	–	–	–	–	–	–	–	–	–
**10**	1.25	1.05	0.57	0.79	8.56	8.33	5.56	0.52	0.49	9.67	7.33
**11**	1.70	0.60	0.45	0.87	8.00	9.00	4.60	0.23	1.14	10.00	4.40
**12**	–	–	–	–	–	–	–	–	–	–	–
**Sample *M***	**1.18**	**0.96**	**0.77**	**0.90**	**7.25**	**7.04**	**5.81**	**0.82**	**0.81**	**8.71**	**6.74**

ED, Euclidean distance; *M*, mean; *p*, participants; -, not applicable; MBU, mother and baby unit.

Mean ranked rating score ranged from 1 to 10, with 1 = the closest positive pole of the construct (preferred) is applied to the element and 10 = the closest negative pole of the construct (non-preferred) is applied to the element. Participants shaded in grey did not complete the discharge repertory grid.

**Table 5:** Changes to the Euclidean distances (standardised) and mean ranked ratings used to calculate self-esteem, self-perception, and construal of non-self elements between admission and discharge.

*p*	Self-esteem				Self-perception			Construal of non-self elements			
	ED: actual self as mother and ideal self as mother	ED: actual self and ideal self	ED: self before becoming a mother and ideal self	ED: self before becoming a mother and actual self	Mean ranked position of actual self	Mean ranked position of actual self as mother	Mean ranked position of self before becoming a mother	ED: actual self as mother and other mother on MBU	ED: actual self as mother and friend who is a mother	Mean ranked position of other mother on MBU	Mean ranked position of friend who is a mother
**1**	–	–	–	–	–	–	–	–	–	–	–
**2**	–	–	–	–	–	–	–	–	–	–	–
**3**	−0.51	−1.17	−0.11	0.09	−4.18	−4.91	2.18	1.16	0.16	0.46	1.45
**4**	0.26	−0.12	0.14	−0.49	−0.54	−1.46	2.08	0.05	−0.54	−1.07	3.07
**5**	−0.22	−0.46	0.05	−0.24	−1.14	−1.86	0.71	0.23	−0.10	1.43	0.78
**6**	0.30	0.06	0.06	0.89	−0.70	0.30	−0.60	0.20	0.26	−0.70	0.20
**7**	−0.27	−0.46	−0.32	−0.08	−0.42	0.00	0.41	−0.49	−0.38	1.25	1.00
**8**	−0.34	−0.17	0.16	0.42	−0.70	−1.70	-1.6	0.37	-0.10	1.00	0.50
**9**	–	–	–	–	–	–	–	–	–	–	–
**10**	−0.24	−0.93	−0.31	−0.43	−1.00	−1.11	1.00	0.16	−0.09	1.67	0.33
**11**	0.34	-0.68	−0.20	−0.56	−1.40	−0.40	0.80	−0.18	0.29	1.90	-1.60
**12**	–	–	–	–	–	–	–	–	–	–	–
**Sample *M***	**−0.14**	**−0.40**	**−0.09**	**0.04**	**−1.46**	**−1.41**	**−0.26**	**0.23**	**−0.05**	**0.91**	**0.81**
**Significant change based on W**	**Non-significant** **(p** = .**05)**	**Significant** **(p** = .**05)**	**Non-significant** **(p** = .**05)**	**Non-significant** **(p** = .**05)**	**Significant** **(p** = .**05)**	**Significant** **(p** = .**05)**	**Non-significant** **(p** = .**05)**	**Non-significant** **(p** = .**05)**	**Non-significant** **(p** = .**05)**	**Non-significant** **(p** = .**05)**	**Non-significant** **(p** = .**05)**

ED, Euclidean distance; *M*, mean; *p*, participants; MBU, mother and baby unit; W, Wilcoxon signed-rank test.

Participants shaded in grey did not complete the discharge repertory grid.

Seven of the eight participants in this sample showed an improvement in their self-esteem during their MBU admission (sample *M* Euclidean distance change = −0.40, *Z* = 1, *p* = .05). This improvement was not observed for one participant, whose self-esteem appeared to decrease over time. Self-esteem as a mother also improved from admission to discharge for five participants, illustrated through a reduction in the Euclidean distance between the *actual current self as mother* and the *ideal self as mother* (sample *M* Euclidean distance change = −0.14, *Z* = 14.5, *p* = n.s.). However, a conflicting pattern of change in construing was observed in three participants, who demonstrated a reduction in their self-esteem as a mother during their MBU admission. It is important to emphasise that despite showing an improvement in their self-esteem as a mother over time, one participant in particular continued to construe extreme dissimilarity between the *actual current self as mother* and the *ideal self as mother* at discharge (i.e., a Euclidean distance of 1.35 between these two elements). Despite the improvements in self-esteem in general and in the maternal role, participants tended to continue to experience low self-esteem in general and in their maternal role at discharge from the MBU, as illustrated by dissimilarity between the *actual current self* and the *ideal self* (sample *M* Euclidean distance = 0.96) and the *actual current self as mother* and the *ideal self as mother* (sample *M* Euclidean distance = 1.18).

Participants construed themselves more positively at discharge, both themselves generally (sample *M* ranked position along construct poles change = −1.46, *Z* = 0, *p* = .05) and themselves as a mother (sample *M* ranked position along construct poles change = −1.41, *Z* = 1, *p* = .05), relative to admission. This pattern of holding a more positive self-perception was observed in all participants, with the exception of two participants’ views of the *actual current self as mother*, when no or very small changes to the mean ranked position of element along construct poles were observed. However, most of the eight participants continued to construe the *actual current self* (sample *M* ranked position along construct poles = 7.25) and the *actual current self as mother* (sample *M* ranked position along construct poles = 7.04) fairly negatively, suggesting participants persisted to hold a negative self-perception at discharge.

Participants generally continued to construe the *self before becoming a mother* (sample *M* ranked position along construct poles = 5.81) positively, relative to current versions of the self. An exemption to this was in the instance of one participant who construed the *actual current self* more positively than the *self before becoming a mother* at discharge. The same participant also construed the *actual current self as mother* more positively than the *self before becoming a mother*, alongside another participant. Six participants were observed to construe the *self before becoming a mother* more negatively at discharge relative to admission (sample *M* ranked position along construct poles change = −0.26, *Z* = 8, *p* = n.s.). *Self before becoming a mother* was construed more positively than a *friend who is a mother* by all participants except two. One of these two participants was also the only participant who construed the *self before becoming a mother* more negatively than the *other mother on the MBU* at discharge. Participants generally construed the *self before becoming a mother* as more similar to the *ideal self* (sample *M* Euclidean distance = 0.77) than the *actual self* (sample *M* Euclidean distance = 0.90) at discharge; however, three participants did not demonstrate this pattern of construing.

At discharge, participants appeared to construe the *actual current self as mother* as slightly more similar to a *friend who is a mother* (sample *M* Euclidean distance = 0.81) than the *other mother on the MBU* (sample *M* Euclidean distance = 0.82), although three participants did not. For a different participant, there was no difference in the conceptual distance between the *actual current self as mother* and a *friend who is a mother* and the *actual current self as mother* and the *other mother on the MBU*. Overall, the conceptual distance between the *actual current self as mother* and a *friend who is a mother* was observed to decrease during MBU admission (sample *M* Euclidean distance change = −0.05, *Z* = 15, *p* = n.s.). Differences to this pattern of change in construing were observed in three participants, with these participants construing themselves as increasingly dissimilar to a *friend who is a mother* over time. Despite an increase in conceptual distance between the *actual current self as mother* and a *friend who is a mother* during admission, one of these three participants did construe the *actual current self as mother* and a *friend who is a mother* as more similar than the *actual current self as mother* and the *other mother on the MBU* at discharge. Except for two participants, participants construed the *actual current self as mother* and the *other mother on the MBU* as increasingly dissimilar during admission (sample *M* Euclidean distance change = 0.23, *Z* = 10, *p* = n.s.). These two participants construed themselves as increasingly alike the *other mother on the MBU* at discharge, relative to how this element pair were construed at admission.

Overall, participants continued to construe a *friend who is a mother* (sample *M* ranked position along construct poles = 6.74) relatively positively, and seven participants construed a *friend who is a mother* more positively than the *other mother on the MBU* (sample *M* ranked position along construct poles = 8.71); only one participant did not demonstrate this pattern. Six participants construed the *other mother on the MBU* most negatively, when comparing against the *actual current self as mother*, the *actual current self*, and a *friend who is a mother*. According to the sample means, participants construed a *friend who is a mother* more positively than the *actual current self as mother* (sample *M* ranked position along construct poles = 7.04) at discharge; however, half of the participants construed the *actual current self as mother* most positively, in comparison with the *actual current self* and both *non-self* elements. Only one participant construed the *actual current self as mother* and the *actual current self*, more negatively than both *non-self* elements at discharge. Generally, participants construed the *non-self* elements, a *friend who is a mother* (sample *M* ranked position along construct poles change = 0.81, *Z* = 7, *p* = n.s.), and the *other mother on the MBU* (sample *M* ranked position along construct poles change = 0.91, *Z* = 6, *p* = n.s.), more negatively over their MBU admission. However, variations to this pattern of change in construing were observed in two participants, who construed the *other mother on the MBU* more positively during admission, and one different participant who construed a *friend who is a mother* more positively over time.

#### 3.3.3 Construal profiles of mother

Participants appeared to fall into one of two possible groups of changes in construing during MBU admission, which were termed in this study as *Profiles*. However, it is important to highlight that some participants appeared to show changes in construing consistent with both profiles. Profile 1 was characterised by an improvement in self-esteem, both in general and in the maternal role. These participants construed the *actual current self as mother* more positively than the *other mother on the MBU* and often more positively than a *friend who is a mother*, during their MBU admission. At discharge, a *friend who is a mother* continued to be construed more positively than the *other mother on the MBU.* These participants construed greater similarity between the *actual current self as mother* and a *friend who is a mother*, in comparison with the *actual current self as mother* and the *other mother on the MBU* at discharge. While subtle deviations from this overall profile were present, these changes in construing seemed to occur for five of the eight participants. Participants who showed changes in construing in line with profile 1 typically had experiences including a supportive network of family and friends who visited the ward regularly. These participants readily engaged in the therapeutic offer on the ward and were able to follow a graded discharge plan. For most of these participants, this was their first inpatient psychiatric admission and all of these participants were admitted to the MBU informally.

Profile 2 was characterised by a reduction in self-esteem, specifically in the maternal role. A *friend who is a mother* continued to be construed more positively than the *actual current self as a mother* and the *other mother on the MBU*; both were construed negatively. These participants construed the *actual current self as mother* as increasingly similar to the *other mother on the MBU*, compared with a *friend who is a mother*, over time. Two participants demonstrated changes in construing consistent with this profile, and in most aspects, a third participant did. Participants who exhibited changes in construing in line with profile 2 typically had experiences including a ‘lonely,’ ‘critical,’ and ‘unsupportive’ living and/or family environment, high economic deprivation, social services involvement and were admitted to the MBU under a section of the Mental Health Act (78). These participants often had a complex mental health history, perceived their mental health condition as highly impairing, and had past psychiatric inpatient admissions. Furthermore, for these participants their admission meant they were more geographically isolated from family and/or friends resulting in a difficulty to experience regular visits from family and partners or follow a discharge plan that included initial brief visits home, which progressed into longer home visits.

One aspect of change in construing that presented in both profiles was the movement towards a more positive self-perception in general and as a mother. Positive self-esteem and a positive self-perception are often understood to hold a nuanced relationship; however, these findings suggested that there were intricate differences in what, when, and how self-esteem and self-perception were influenced and shaped during an MBU admission.

#### 3.3.4 Construing at admission and discharge

The mean percentage of variance accounted for by the first principal component at admission was high (sample *M* eigenvalue = 81.84%, SD = 13.27; see **Table 6**), with 11 of the 12 participants recording eigenvalues greater than 70%, which is indicative of ‘tight’ construing 46,51,78]. Only one participant presented with an eigenvalue indicative of ‘loose’ construing (eigenvalue = 48.72%).

**Table 6:** Construing reflected through percentage variance accounted for by the first principal component produced by the PCA.

p	Eigenvalue: percentage variance accounted for by the first principal component (admission)	Eigenvalue: percentage variance accounted for by the first principal component (discharge)	Change to eigenvalue: percentage variance accounted for by the first principal component from admission to discharge
**1**	94.88%	–	–
**2**	81.39%	–	–
**3**	79.62%	66.30%	Decrease
**4**	48.72%	41.45%	Decrease
**5**	95.43%	87.87%	Decrease
**6**	82.64%	62.55%	Decrease
**7**	71.36%	88.23%	Increase
**8**	83.39%	73.87%	Decrease
**9**	88.22%	–	–
**10**	97.10%	93.12%	Decrease
**11**	86.18%	88.00%	Increase
**12**	73.14%	–	–
**Sample *M***	**81.84%**	**75.17%**	**Decrease**
**Significant change based on W**	**-**	**-**	**Non-significant** **(p** = .**05)**

*M*, mean; *p*, participants; W, Wilcoxon signed-rank test; PCA, principal component analysis.

An eigenvalue greater than 70% is generally considered to be indicative of ‘tight’ construing (Blagden & Needs, 2022; Fransella et al., 2004; Winter, 1992). Participants shaded in grey did not complete the discharge repertory grid.

**Figure 2:**
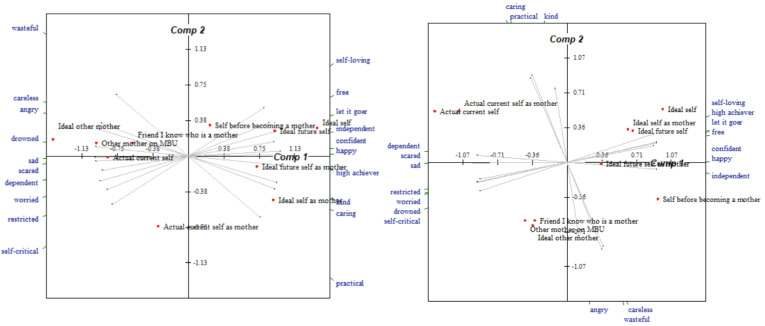
Bi-plots of a participant’s repertory grids completed at admission and discharge respectively, who displayed ‘loosening’ of the conceptual system during their MBU admission.

**Figure 3:**
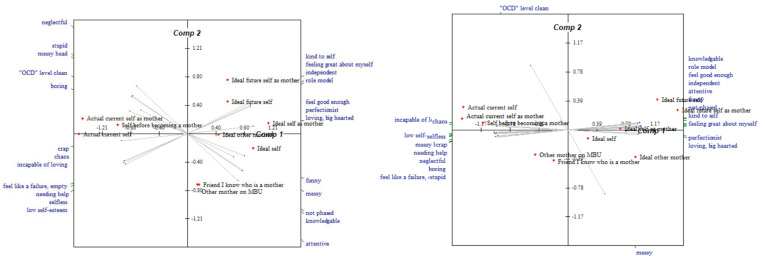
Bi-plots of a participant’s repertory grids completed at admission and discharge respectively, who displayed ‘tightening’ of the conceptual system during their MBU admission.

Construing appeared to ‘loosen’ for most participants between admission and discharge, as shown through a decrease in eigenvalues (sample *M* eigenvalue change = −6.67%, *Z* = 8, *p* = n.s.); however, overall, construing remained ‘tight’ at discharge (sample *M* eigenvalue = 75.17%, SD = 17.71). Of the eight participants who completed both admission and discharge repertory grids, six participants displayed ‘loosening’ of their conceptual system over the course of their admission. Of these six participants, three participants continued to demonstrate an eigenvalue greater than 70%. Comparatively, two participants showed an increase in eigenvalue between admission and discharge grids, suggesting construing became ‘tighter’ over time. To illustrate the ‘loosening’ and ‘tightening’ of conceptual structure exhibited by two participants respectively, the relevant PCAs are illustrated in **Figures 2**, **3**.

Of the six participants who displayed ‘loosening’ of their conceptual system over time, five of these participants also demonstrated changes in construing that were more in keeping with profile 1 discussed in *3.3.3 Construal profiles of mothers* during their MBU admission. The two participants who showed ‘tightening’ of their conceptual structure were participants whose pattern of construing changed during admission in a manner more aligned with the second profile.

#### 3.3.5 Construing and psychological distress

Of the eight participants who completed discharge repertory grids, seven participants (87.50%) who showed a reliable improvement in psychological distress as captured by the CORE-OM (i.e., a decrease of >0.5 in *mean rating applied across all sub-scales*) also showed an improvement in their self-esteem in general. The participant (12.50%) who did not show an improvement in psychological distress (i.e., a non-reliable increase of <0.5 in *mean rating applied across all sub-scales*) showed a reduction in overall self-esteem over time. Of the six participants (75.00%) who displayed ‘loosening’ of their conceptual system, five of these participants (62.50%) showed an improvement in psychological distress over time. Both participants (25.00%) who displayed ‘tightening’ of their conceptual system also displayed an improvement in psychological distress.

#### 3.3.6 Content of constructs at admission

All 12 repertory grids developed at admission were subjected to content analysis. Overall, 129 constructs were elicited (see **Table 7**). Most constructs were classified in the construct categories, *Personal* (*n* = 37), *Emotional* (*n* = 35), and *Relational* (*n* = 28), indicating that the emphasis for participants was on their symptom experience, relational style, and personal characteristics. Commonly elicited constructs within the *Personal* category were those related to confidence, such as ‘self-confident–low self-esteem’. Several participants who elicited this type of construct described ‘confidence’ to fall at the preferred end of the construct pole. They described this person as someone who was self-assured, often in relation to their capabilities as a mother, and frequently opposed this to a person who was ‘self-critical’. All participants elicited constructs categorised as *Emotional* and for many, this related to their symptom experiences at the time of admission; for instance, “happy–sad” and “calm–anxious”. Almost all participants elicited at least one construct within the *Relational* category in reference to the mother–infant relationship (e.g., ‘willing and attentive to child’s needs - avoiding giving care’), which could have suggested that participants regarded this relationship as focal and important at admission to the MBU.

**Table 7:** Results of the content analysis of constructs using the CSPC.

Construct category	Number of constructs (%)	Examples
**Moral**	15 (11.63%)	‘caring-cruel’
**Emotional**	35 (27.13%)	‘irritable–calm’, ‘happy–sad’, ‘relaxed–anxious’
**Relational**	28 (21.71%)	‘wanted–isolated’, ‘sociable–go inwards’
**Personal**	37 (28.68%)	‘low self-esteem–feeling great about myself’, ‘self-confident–no confidence at all’,
**Intellectual/operational**	9 (6.98%)	‘knowledgeable–stupid’, ‘multitasker–difficulty concentrating’
**Values/interests**	1 (0.78%)	‘creative–clinical’
**Existential** *(supplementary)*	4 (3.10%)	‘existing–living’
**Concrete** *(supplementary)*	0 (0.00%)	–
**Total**	**129**	–

-, non-applicable.

CSPC, Classification System for Personal Construct analysis categories (Feixas et al., 2002).

Some but not all participants also elicited constructs that were classified into the *Moral* (*n* = 15) and *Intellectual/Operational* (*n* = 9) categories. Many of the constructs classified as *Intellectual/Operational* related to being knowledgeable about the practical skills and abilities necessary for being a mother. Given that the construct category *Intellectual/Operational* had fewer constructs relative to other categories, it could be inferred that participants considered personal characteristics and the emotional and relational experience as more important over possessing skills, ability, and knowledge at an intellectual level at the point of admission; however, this inference is only notional and would necessitate further investigation for validation.

Only one construct was noted in the category of *Values/Interests*, and four constructs fell in the supplementary category of *Existential*, all being related to lacking purpose or feeling empty. All participants who elicited constructs classified in the *Existential* category had a primary diagnosis of postnatal depression. No constructs were elicited which fell within the *Concrete* category; however, this might have been due to participants being discouraged from providing similarities or differences based on physical characteristics during construct elicitation.

The variability in the constructs elicited demonstrated the idiosyncrasy of the terms used by participants to construe elements. A detailed description was gathered from participants during construct elicitation because the same construct was sometimes categorised differently for participants depending on the meaning attributed. For instance, several participants elicited the word ‘relaxed’ for the preferred end of the construct pole. For some participants, ‘relaxed’ related to their emotional experience and was opposed to feeling ‘anxious’. Some participants described ‘relaxed’ as a personal characteristic, someone who was easy-going and open-minded. ‘Relaxed’ for one participant was used to describe her relational experience and used to contrast the relational experience of feeling “threatened”.

## 4 Discussion

This is the first study to use RGT to investigate change in construal shown by mothers over the course of an MBU admission. Thereby, this exploratory study provided new insights into the role of MBUs in supporting mental health recovery for women during the perinatal period. By repeating the RGT at admission and discharge, changes in a mother’s construal during an MBU admission were revealed: RGT data showed that most participants demonstrated an improvement in their psychological well-being during an MBU admission, evidenced by an improvement in their self-esteem in general and in their maternal role, and the movement towards a more positive self-perception. MBU admission supported most participants to begin to construe themselves as more like a mother residing in the community, who they had construed positively, and less like a mother with a mental health condition that necessitated an inpatient admission, who they had construed negatively. These findings suggested that the MBU admission appeared to fulfil the stipulations of MBUs as denoted by the UK’s National Institute for Health and Care Excellence (30), with regard to supporting maternal mental health and potentially strengthening the mother–infant relationship by improving self-esteem in the maternal role. Furthermore, the discovery of these changes in the current study corroborated the findings of previous studies that have used alternative methodologies to illustrate that MBU admission improved maternal clinical symptoms (38–40). However, the RGT used in this study revealed particularly novel findings about the impact of MBU admission on mothers: most participants admitted to the ward exhibited extremely ‘tight’ construing which became ‘looser’ over time. This finding inferred that an MBU admission and associated psychiatric and psychological treatment supported most participants to move towards less ‘extreme’ construing, which Kelly (47) postulated is indicative of better mental health.

Given that the MBU admission was defined as the therapeutic intervention in this study, the findings of this current study offer validation that MBU admission serves as a helpful form of therapeutic intervention for most mothers, irrespective of diagnosis, length of admission, or specific psychological, psychiatric, or nursing input. Indeed, there are similarities in the changes in construing observed in participants who experienced positive outcomes in this present study and people with a range of mental health conditions, who experienced positive outcomes following other forms of therapeutic intervention (60, 79, 80).

The present study found that most participants exhibited a decrease in the conceptual distance between the *actual current self as mother* and the *ideal self as mother* over time, thus substantiating the notion that if mothers experience a decrease in the discrepancy between motherhood ideals and lived reality, they could experience a reduction in a range of psychological difficulties (7, 13–17). However, further research is required to verify these tentative observations.

As mentioned above, most participants experienced positive psychological changes in relation to their self-esteem, self-perception, how they construed the self in relation to others, and the construing process. However, the present study also revealed that a few participants did not experience the aforementioned positive psychological changes, highlighting that not all mothers experienced positive changes in construing during their MBU admission. Speculative observations arose when reflecting on the possible commonalities in the contextual experiences of participants who displayed similar changes in construing during their MBU admission and led to the identification of two potential profiles of changes to conceptual structure shown by participants. However, it must be emphasised that these observations were purely tentative speculations and were based on a small sample of eight participants; hence, further research to substantiate these observations is imperative.

Termed as profile 1 in this current study, it seemed that participants who experienced positive psychological changes during their MBU admission were more likely to present with a less complex mental health history, experienced regular visits from their family and partner, and had access to a supportive and stable wider support network at discharge. Conversely, termed as profile 2, participants who were vulnerable to experiencing fewer positive psychological changes were more likely to present with multiple previous admissions to psychiatric inpatient care, fewer experiences of sensitive and attuned social support both during admission and at discharge, and economic deprivation and were more likely to perceive themselves as more impacted by their mental health condition.

Interestingly, these tentative observations from the current study regarding the interplay between positive psychological changes and contextual experiences appeared to be reflective of some of the findings in the wider literature on mental health recovery outcomes. Within other samples of people experiencing mental health conditions, poorer mental health recovery outcomes have been associated with poorer perceived social support and greater loneliness (81–84), greater perceived mental health impairment (85), higher economic deprivation (86), more frequent presentation to health services (87), and higher complexity and/or comorbidity of mental health conditions (88).

### 4.1 Clinical implications

The findings of this study point towards several important implications. During the assessment sessions, almost all participants expressed that they had experienced heightened anxiety regarding being admitted to an MBU for a plethora of reasons including apprehension about inpatient psychiatric care and separation from family and other children. As most participants underwent a positive shift in self-perception and growth in self-esteem during their admission, facets that are associated with improvement in psychiatric clinical symptomology and psychological well-being (20, 21), these findings may reassure mothers of the potential benefits associated with such an admission. Furthermore, with the knowledge that an MBU admission appeared to provide most mothers with positive therapeutic benefits, family members and partners could be encouraged to actively seek this form of support when it is indicated.

The findings also highlighted potential targets for psychological intervention. Most participants displayed a ‘loosening’ of conceptual structure over time, suggesting that MBU admission supported most participants to increase their cognitive flexibility: a concept linked with improved psychological well-being (47). Therefore, mothers admitted to an MBU who initially present with extremely ‘tight’ construing may benefit from MBU staff supporting them to develop a more ‘moderate’ and adaptive construing process. It should be noted that ‘loosening’ in construing was observed across participants who would have received varied therapeutic support during their admission, indicating that ‘loosening’ in construing did not appear to be related to one specific intervention. Furthermore, most participants in this current study, who experienced positive psychological changes during their MBU admission, exhibited changes to their conceptual structure indicative of forming the motherhood identity, i.e., a decrease in the conceptual distance between the *actual current self as mother* and the *ideal self as mother*. Therefore, MBU staff should explore the personalised and internalised societal motherhood ideals held by the service user, and support mothers further to integrate these with their actual experience of motherhood to promote positive change to psychological well-being.

Positive changes in construing were observed for most participants irrespective of mental health diagnoses and receiving personalised, and hence, varied therapeutic interventions. This finding suggested that MBU staff can be reassured that the overall therapeutic offer of an MBU, although tailored for each mother, appears to contribute to largely positive changes in construing for most mothers. Therefore, the present findings emphasised the efficacy of the current transdiagnostic and multidisciplinary treatment model implemented by the MBUs in supporting the diverse needs of mothers. However, awareness of the two possible profiles of changes in construing for mothers could inform how MBU staff approach care planning for certain mothers, particularly mothers presenting with contextual experiences that appear consistent with participants who experienced fewer positive changes in this current study (profile 2). MBU staff should have reassurance that even modest change during an admission might prove beneficial for these mothers. For these mothers, discharge may present as more challenging time; hence, MBU staff should ensure that these mothers receive more intensive input from community perinatal mental health teams alongside identified alternative avenues of community-based support at discharge, such as support from social services and the volunteer sector, to encourage a successful transition back to the community and reduce the risk of social isolation or hospital readmission.

While most participants showed psychological improvements during their admission, the current findings showed that several dimensions of conceptual structure remained in the realms of ‘problematic’ at discharge. As all participants presented with an ongoing need for psychological intervention beyond MBU admission, the current findings emphasised the necessity for continued psychological intervention for all mothers discharged from an MBU, provided through community perinatal mental health teams (31). Therefore, the close collaboration between community and inpatient services is indicated to be best practice (31), to build on the positive outcomes achieved through an MBU admission and facilitate effective, long-term psychological change for mothers.

Given the importance of family and partner support for participants in this current study, the proximity of an MBU to a mother’s support network could be crucial. Despite the UK having 19 MBU facilities, with NHS England declaring in 2019 that all 44 regions have access to this specialist service (NHS England, 2019), the provision of MBUs continues to vary considerably. Geographical disparities in MBUs persist in the UK (89), leading to out-of-area admissions for mothers, which could have negative consequences on their mental health and on services including increased length of stay, greater subsequent contact with mental health services and a greater average cost (90). While the NHS long-term plan outlined significant financial investment for perinatal services (91), further investment in additional MBUs, guided by geographical location, might be necessary to enhance perinatal services for the most seriously unwell women. This investment could support a more therapeutic admission by facilitating more regular visits from family and partners.

### 4.2 Strengths, limitations, and future research

The use of the RGT in this present study was a notable strength over alternative methodologies because of reasons highlighted in the introduction. Furthermore, the RGT bridged the gap between qualitative and quantitative methodologies, because both qualitative and quantitative data were yielded, meaning both approaches to analysis were viable for this study (48, 54, 92). Implementing the RGT meant a participant’s subjective experience was measured objectively, thus maintaining statistical rigor (52, 54). As eight participants completed both assessment sessions, the sample size was comparable with previous RGT studies which have implemented the RGT across different time points (59, 62, 63) and power calculations were not required because a representative sample was a greater priority than generalisability.

However, as some limitations must also be discussed, suggestions for future research will be outlined. Efforts were made to generate a larger recruitment pool by sourcing participants from two MBUs. Before the study findings can be confidently transferred to other MBU contexts, replication on a larger scale, incorporating MBUs spanning a more expansive geographical area is necessary. Regarding the recruitment of the study sample, selection and attrition biases should be considered. Initially, like most research studies, the main researcher was not permitted by ethics to approach participants directly; hence, eligibility was assessed by MBU staff, who were asked to evaluate a mother’s capacity to consent. This approach could be deemed a subjective judgement by staff (93), meaning selection bias could have been present. Staff expressed greater uncertainty in assessing eligibility for psychosis and related disorders, suggesting certain mental health diagnoses might have been more vulnerable to exclusion. Furthermore, several mothers declined to take part in the study at various recruitment stages for several reasons (see **Figure 1**), meaning consideration must be given for potential characteristics, traits, or experiences that might have influenced initial willingness to participate or subsequent drop-out. This pattern of participant retention and drop-out raised concerns regarding attrition bias, suggesting that there might have been differences in the characteristics or experiences of mothers who withdrew from the study and those who completed the second repertory grid.

Another notable limitation was the lack of ethnic diversity among participants, with 91.67% identifying as White British. Resource limitations prevented the use of translation services, and four mothers (3.28%) who were not proficient in English were not approached for this reason (see **Figure 1**). As self-construal is understood to be shaped by race and culture (94, 95), the understanding gained about maternal construal at MBU admission and discharge might be limited to the experience of White British mothers, therefore potentially hindering the transfer of study findings to other groups of mothers. To increase the representativeness of mothers nationally, future research should strive to recruit a more ethnically diverse sample.

Changes to conceptual structure appeared to begin during an MBU admission; however, assessing for substantial and enduring changes in construing following MBU admission was not feasible. All discharge assessment sessions were completed on the day of or within 1 week of discharge, prohibiting confirmation of whether the changes observed were maintained once discharged. Future research aimed at assessing the longevity of changes achieved through an MBU admission and the continued trajectory of conceptual structure changes once mothers return to the community following a significant period of time would be valuable. This research would enhance our understanding of the longer-term benefits of MBU admission and the interplay between conceptual structure and psychological well-being for mothers during the perinatal period. Finally, the observations made between the possible commonalities in the contextual experiences of participants who displayed similar changes in construing during an MBU admission were speculative. To draw robust conclusions regarding the probable multifaceted nature of the contributory factors to the changes in construing observed in this sample of participants, further research exploring this particular aspect with a much larger sample size would be necessary.

A further possible limitation of the current study, even allowing for the inherent strengths of the idiographic RGT methodology, is the lack of any clinical or statistical controls. The repeated assessment was designed to examine change over the course of an inpatient admission, and it was intended that participants would effectively act as their own control in line with other small N research designs. Furthermore, it would be difficult to identify an appropriate control group because the study was designed to look at the nature of changes, if any, in construing over the course of an MBU admission rather than any specific clinical interventions that might have been received while on the MBU. Taking this into account, future studies might look at identifying comparison groups, such as mothers receiving treatment in the community.

### 4.3 Conclusions

For the first time, this study shed light on the changes in construing exhibited by mothers during an MBU admission. Most mothers displayed an improvement in psychological well-being, demonstrated by improvements in their self-esteem in general and in their maternal role, movement towards a more positive self-perception, increased construed similarity between the self and positively construed others, and a ‘loosening’ in construing. While findings emphasised the worth of MBU provision for improving maternal mental health for most mothers, variations in the changes to conceptual structure exhibited were observed and two possible profiles of changes emerged. Implications of this research extend to service users, their families, MBU clinicians, and stakeholders in perinatal mental health care. Broadening investigations encompassing other MBU contexts nationwide and diversifying the representation of mothers beyond White British is recommended.

## Data Availability

The raw data supporting the conclusions of this article will be made available by the authors, without undue reservation.

## References

[1] Mercer RT. Becoming a mother versus maternal role attainment. J Nurs Scholarsh. (2004) 36:226–32. doi: 10.1111/j.1547-5069.2004.04042.x 15495491

[2] Lorén H Weineland S Rembeck G. Facing a new life-The healthy transition to motherhood: What individual and environmental factors are needed? A phenomenological-hermeneutic study. Midwifery. (2024) 130:103917. doi: 10.1016/j.midw.2024.103917 38232668

[3] NHS England. The Perinatal Mental Health care pathways (2018). Available online at: https://www.england.nhs.uk/publication/the-perinatal-mental-health-care-pathways/ (Accessed 2024 Mar 19).

[4] Laney EK Hall MEL Anderson TL Willingham MM. Becoming a mother: The influence of motherhood on women’s identity development. Identity. (2015) 15:126–45. doi: 10.1080/15283488.2015.1023440

[5] Arnold-Baker C. Introduction: the existential crisis of motherhood. In: Arnold-Baker C, editor. The existential crisis of motherhood. Palgrave Macmillan, Cham (2020). p. 3–16.

[6] Wills L. (2017). The effect of the relational self-construal on the wellbeing of mothers of preschool aged children, in: higher degree by research conference, , Jun 22-23.

[7] Liss M Schiffrin HH Rizzo KM. Maternal guilt and shame: The role of self-discrepancy and fear of negative evaluation. J Child Fam Stud. (2013) 22:1112–9. doi: 10.1007/s10826-012-9673-2

[8] Staneva A Wittkowski A. Exploring beliefs and expectations about motherhood in Bulgarian mothers: a qualitative study. Midwifery. (2013) 29:260–7. doi: 10.1016/j.midw.2012.01.008 22341091

[9] Williamson T Wagstaff DL Goodwin J Smith N. Mothering ideology: A qualitative exploration of mothers’ perceptions of navigating motherhood pressures and partner relationships. Sex Roles. (2023) 88:101–17. doi: 10.1007/s11199-022-01345-7 PMC9765384 36568897

[10] Zhou M. Motherhood, employment, and the dynamics of women’s gender attitudes. Gend Soc. (2017) 31:751–76. doi: 10.1177/089

[11] Choi P Henshaw C Baker S Tree J. Supermum, superwife, supereverything: performing femininity in the transition to motherhood. J Reprod Infant Psychol. (2005) 23:167–80. doi: 10.1080/02646830500129487

[12] Lupton D. [amp]]lsquo;A love/hate relationship’: the ideals and experiences of first- time mothers. J Sociol. (2000) 36:50–63. doi: 10.1177/144078330003600104

[13] Rossouw P Lazarus K. Mothers’ expectations of parenthood: the impact of prenatal expectations on self-esteem, depression, anxiety, and stress post birth. Int J Neuropsychotherapy. (2015) 3:102–23. doi: 10.12744/ijnpt.2015.0102-0123

[14] Law NK Hall PL Cheshire A. Common negative thoughts in early motherhood and their relationship to guilt, shame and depression. J Child Fam Stud. (2021) 30:1831–45. doi: 10.1007/s10826-021-01968-6

[15] Sonnenburg C Miller YD. Postnatal depression: The role of “good mother” ideals and maternal shame in a community sample of mothers in Australia. Sex Roles. (2021) 85:661–76. doi: 10.1007/s11199-021-01239-0

[16] Miller T. is this what motherhood is all about?”: Weaving experiences and discourse through transition to first-time motherhood. Gend Soc. (2007) 21:337–58. doi: 10.1177/0891243207300561

[17] Sutherland J-A. Mothering, guilt and shame: Mothering, guilt and shame. Sociol Compass. (2010) 4:310–21. doi: 10.1111/j.1751-9020.2010.00283.x

[18] Cutrona CE Troutman BR. Social support, infant temperament, and parenting self-efficacy: A mediational model of postpartum depression. Child Dev. (1986) 57:1507. doi: 10.2307/1130428 3802975

[19] Coyne SM McDaniel BT Stockdale LA. Do you dare to compare?” Associations between maternal social comparisons on social networking sites and parenting, mental health, and romantic relationship outcomes. Comput Hum Behav. (2017) 70:335–40. doi: 10.1016/j.chb.2016.12.081

[20] Hutchinson J Cassidy T. Well-being, self-esteem and body satisfaction in new mothers. J Reprod Infant Psychol. (2022) 40:532–46. doi: 10.1080/02646838.2021.1916452 33877938

[21] Kim S Bang K-S Lee G Lim J Jeong Y Song MK. Stressors and stress responses of unmarried mothers based on Betty Neuman’s systems model: An integrative review. Child Health Nurs Res. (2020) 26:238–53. doi: 10.4094/chnr.2020.26.2.238 PMC8650940 35004468

[22] Dimitrovsky L David-fuchs M Itskowitz R. Self-acceptance and attitudes towards pregnancy and femininity among primiparae. J Reprod Infant Psychol. (1989) 7:203–10. doi: 10.1080/02646838908403595

[23] American College of Obstetricians and Gynaecologists. ACOG committee opinion no. 757: Screening for perinatal depression. Obstet Gynecol. (2018) 132:e208–12. doi: 10.1097/AOG.0000000000002927 30629567

[24] Howard LM Khalifeh H. Perinatal mental health: a review of progress and challenges. World Psychiatry. (2020) 19:313–27. doi: 10.1002/wps.20769 PMC7491613 32931106

[25] Howard LM Molyneaux E Dennis C-L Rochat T Stein A Milgrom J. Non-psychotic mental disorders in the perinatal period. Lancet. (2014) 384:1775–88. doi: 10.1016/S0140-6736(14)61276-9 25455248

[26] Jones I Chandra PS Dazzan P Howard LM. Bipolar disorder, affective psychosis, and schizophrenia in pregnancy and the post-partum period. Lancet. (2014) v384:1789–99. doi: 10.1016/S0140-6736(14)61278-2 25455249

[27] Munk-Olsen T Maegbaek ML Johannsen BM Liu X Howard LM di Florio A. Perinatal psychiatric episodes: a population-based study on treatment incidence and prevalence. Transl Psychiatry. (2016) 6:e919. doi: 10.1038/tp.2016.190 27754485 PMC5315550

[28] O’Hara MW Wisner KL. Perinatal mental illness: definition, description and aetiology. Best Pract Res Clin Obstet Gynaecol. (2014) 28:3–12. doi: 10.1016/j.bpobgyn.2013.09.002 24140480 PMC7077785

[29] World Health Organization. Guide for integration of perinatal mental health in maternal and child health services. Genève. (2022). https://iris.who.int/bitstream/handle/10665/362880/9789240057142-eng.pdf?sequence=1.

[30] National Institute for Health and Care Excellence. Antenatal and postnatal mental health: clinical management and service guidance (CG192) (2014). London: NICE. Available online at: https://www.nice.org.uk/guidance/cg192/resources/antenatal-and-postnatal-mental-health-clinical-management-and-service-guidance-pdf-35109869806789 (Accessed 2023 Dec 13).

[31] Royal College of Psychiatrists. Perinatal mental health services: recommendations for the provision of services for childbearing women (CR232) (2021). Available online at: https://www.rcpsych.ac.uk/docs/default-source/improving-care/better-mh-policy/college-reports/college-report-cr232—perinatal-mental-heath-services.pdf?msclkid=cdf3d6fdcedd11eca864dad2c20181ec (Accessed 2024 Mar 18).

[32] Royal College of Psychiatrists. PQN quality standards for inpatient perinatal services. Available online at: https://www.rcpsych.ac.uk/docs/default-source/improving-care/ccqi/quality-networks/perinatal/pqn-inpatient-standards—eighth-edition.pdf?sfvrsn=1e827bd_4 (Accessed 2024 Mar 19).

[33] Dowse E Chan S Ebert L Wynne O Thomas S Jones D. Impact of perinatal depression and anxiety on birth outcomes: A retrospective data analysis. Matern Child Health J. (2020) 24:718–26. doi: 10.1007/s10995-020-02906-6 32303935

[34] Kendig S Keats JP Hoffman MC Kay LB Miller ES Moore Simas TA. Consensus bundle on maternal mental health: Perinatal depression and anxiety. Obstet Gynecol. (2017) 129:422–30. doi: 10.1097/AOG.0000000000001902 PMC5957550 28178041

[35] O’Brien J Gregg L Wittkowski A. A systematic review of clinical psychological guidance for perinatal mental health. BMC Psychiatry. (2023) 23:790. doi: 10.1186/s12888-023-05173-1 37904101 PMC10614401

[36] Gillham R Wittkowski A. Outcomes for women admitted to a mother and baby unit: a systematic review. Int J Womens Health. (2015) 7:459–76. doi: 10.2147/IJWH.S69472 PMC4425328 25995650

[37] Connellan K Bartholomaeus C Due C Riggs DW. A systematic review of research on psychiatric mother-baby units. Arch Womens Ment Health. (2017) 20:373–88. doi: 10.1007/s00737-017-0718-9 28332002

[38] Branjerdporn G Hudson C Sheshinski R Parlato L Healey L Ellis A. Evaluation of an inpatient psychiatric mother-baby unit using a patient reported experience and outcome measure. Int J Environ Res Public Health. (2022) 19:5574. doi: 10.3390/ijerph19095574 35564969 PMC9106046

[39] Stephenson LA Macdonald AJD Seneviratne G Waites F Pawlby S. Mother and Baby Units matter: improved outcomes for both. BJPsych Open. (2018) 4:119–25. doi: 10.1192/bjo.2018.7 PMC6020269 29971155

[40] Wright T Stevens S Wouldes TA. Mothers and their infants co-admitted to a newly developed mother-baby unit: Characteristics and outcomes: MBU maternal and infant outcomes. Infant Ment Health J. (2018) 39:707–17. doi: 10.1002/imhj.21742 30339733

[41] Griffiths J Lever Taylor B Morant N Bick D Howard LM Seneviratne G. A qualitative comparison of experiences of specialist mother and baby units versus general psychiatric wards. BMC Psychiatry. (2019) 19:401. doi: 10.1186/s12888-019-2389-8 31842836 PMC6916441

[42] Antonysamy A Wieck A Wittkowski A. Service satisfaction on discharge from a psychiatric mother and baby unit: a representative patient survey. Arch Womens Ment Health. (2009) 12:359–62. doi: 10.1007/s00737-009-0085-2 19575280

[43] Howard LM Trevillion K Potts L Heslin M Pickles A Byford S. Effectiveness and cost-effectiveness of psychiatric mother and baby units: quasi-experimental study. Br J Psychiatry. (2022) 221:628–36. doi: 10.1192/bjp.2022.48 35505514

[44] Wright T Jowsey T Stanton J Elder H Stevens S Wouldes TA. Patient experience of a psychiatric Mother Baby Unit. PloS One. (2018) 13:e0198241. doi: 10.1371/journal.pone.0198241 29847584 PMC5976160

[45] Krumpal I. Determinants of social desirability bias in sensitive surveys: a literature review. Qual Quant. (2013) 47:2025–47. doi: 10.1007/s11135-011-9640-9

[46] Fransella F Bell R Bannister D. A manual for repertory grid technique. 2nd ed. Nashville, TN, USA: John Wiley & Sons (2004).

[47] Kelly GA. The psychology of personal constructs. New York: Norton (1995).

[48] Bourne D Jankowicz DA. The repertory grid technique. In: Ciesielska M Jemielniak D, editors. Qualitative methodologies in organization studies. Springer International Publishing, Cham (2018). p. 127–49.

[49] Böker H Hell D Budischewski K Eppel A Härtling F Rinnert H. Personality and object relations in patients with affective disorders: idiographic research by means of the repertory grid technique. J Affect Disord. (2000) 60:53–9. doi: 10.1016/S0165-0327(99)00161-5 10940448

[50] Taylor PJ Usher S Jomar K Forrester R. Investigating self-concept in self-harm: A repertory grid study: Self-concept and self-harm. Psychol Psychother. (2021) 94 Suppl 2:171–87. doi: 10.1111/papt.12269 32012440

[51] Winter DA. Personal construct psychology in clinical practice: Theory, research and applications. London: Routledge (1992).

[52] Winter DA. Repertory grid technique as a psychotherapy research measure. Psychother Res. (2003) 13:25–42. doi: 10.1093/ptr/kpg005 22475161

[53] Large RG. The use of the role construct repertory grid in studying changes during psychotherapy. Aust N Z J Psychiatry. (1976) 10:315–20. doi: 10.3109/00048677609159518 1071415

[54] Jankowicz D. The easy guide to repertory grids. NJ, USA: Wiley-Blackwell (2003).

[55] Axford S Jerrom DW. Self-esteem in depression: a controlled repertory grid investigation. Br J Med Psychol. (1986) 59:61–8. doi: 10.1111/j.2044-8341.1986.tb02666.x 3964587

[56] Hewstone M Hooper D Miller K. Psychological change in neurotic depression: a repertory grid and personal construct theory approach. Br J Psychiatry. (1981) 139:47–51. doi: 10.1192/bjp.139.1.47 7296190

[57] Makhlouf-Norris F Norris H. The obsessive compulsive syndrome as a neurotic device for the reduction of self-uncertainty. Br J Psychiatry. (1973) 122:277–88. doi: 10.1192/bjp.122.3.277 4696448

[58] Paget A Ellett L. Relationships among self, others, and persecutors in individuals with persecutory delusions: a repertory grid analysis. Behav Ther. (2014) 45:273–82. doi: 10.1016/j.beth.2013.12.001 24491202

[59] McNair L Woodrow C Hare D. Using repertory grid techniques to measure change following dialectical behaviour therapy with adults with learning disabilities: two case studies. Br J Learn Disabil. (2016) 44:247–56. doi: 10.1111/bld.12142

[60] Randal C Bucci S Morera T Barrett M Pratt D. Mindfulness-Based Cognitive Therapy for psychosis: Measuring psychological change using repertory grids: Mindfulness-based cognitive therapy for psychosis. Clin Psychol Psychother. (2016) 23:496–508. doi: 10.1002/cpp.1966 26077540

[61] Vitali D Santini A Sayers L Mospan A. Implicative dilemmas and symptomatology measures: A practice-based evidence study of existential therapy. J Constr Psychol. (2020) 33:351–66. doi: 10.1080/10720537.2019.1592038

[62] Clarke S Pearson C. Personal constructs of male survivors of childhood sexual abuse receiving cognitive analytic therapy. Br J Med Psychol. (2000) 73:169–77. doi: 10.1348/000711200160408 10874477

[63] Madill A Latchford G. Identity change and the human dissection experience over the first year of medical training. Soc Sci Med. (2005) 60:1637–47. doi: 10.1016/j.socscimed.2004.08.035 15652694

[64] Wittkowski A Hare DJ Gillham R. Using the repertory grid technique to explore the experience of compassion by mothers in a mother and baby unit. Midwifery. (2019) 75:24–32. doi: 10.1016/j.midw.2019.04.003 30986691

[65] Health Research Authority. UK policy framework for health and social care research (2021). Available online at: https://www.hra.nhs.uk/planning-and-improving-research/policies-standards-legislation/uk-policy-framework-health-social-care-research/ (Accessed 2024 Mar 19).

[66] Evans J Mellor-Clark F Mar C. CORE: Clinical outcomes in routine evaluation. J Ment Health. (2000) 9:247–55. doi: 10.1080/jmh.9.3.247.255

[67] Evans C Connell J Barkham M Margison F McGrath G Mellor-Clark J. Towards a standardised brief outcome measure: Psychometric properties and utility of the CORE–OM. Br J Psychiatry. (2002) 180:51–60. doi: 10.1192/bjp.180.1.51 11772852

[68] Clinical Outcomes in Routine Evaluation and CST. Information about the CORE-OM (2023). Available online at: https://www.coresystemtrust.org.uk/home/instruments/core-om-information/ (Accessed 2024 Mar 15).

[69] Royal College of Psychiatrists. Framework for routine outcome measures in perinatal psychiatry(CR216) (2018). Available online at: https://www.rcpsych.ac.uk/docs/default-source/improving-care/better-mh-policy/college-reports/college-report-cr216.pdf?sfvrsn=12b1e81c_2 (Accessed 2024 Mar 19).

[70] Barkham M Margison F Leach C Lucock M Mellor-Clark J Evans C. Service profiling and outcomes benchmarking using the CORE-OM: Toward practice-based evidence in the psychological therapies. J Consult Clin Psychol. (2001) 69:184–96. doi: 10.1037//0022-006x.69.2.184 11393596

[71] Reddy PCP. A comparison of triadic and dyadic methods of personal construct elicitation. J Constr Psychol. (1999) 12:253–64. doi: 10.1080/107205399266109

[72] IBM Corporation. IBM SPSS Statistics for Windows, Version 29.0. (2022). Armonk, NY: IBM Corp.

[73] McInnes B. Made to measure: CORE (2018). Available online at: https://therapymeetsnumbers.com/made-to-measure-core/ (Accessed 2024 Mar 26).

[74] Grice JW. Idiogrid: software for the management and analysis of repertory grids. Behav Res Methods Instrum Comput. (2002) 34:338–41. doi: 10.3758/BF03195461 12395549

[75] Slater P. Measurement of intrapersonal space by grid technique: Dimensions of intrapersonal space v. 2. Chichester: John Wiley & Sons (1977).

[76] Slater P. Notes on ingrid 67. London University: Institute of Psychiatry (1967).

[77] Feixas G Geldschläger H Neimeyer RA. Content analysis of personal constructs. J Constr Psychol. (2002) 15:1–19. doi: 10.1080/107205302753305692

[78] UK Government. Mental health act 1983 (2024). Available online at: https://www.legislation.gov.uk/ukpga/1983/20 (Accessed 2024 Mar 21).

[79] Winter D Gournay K Metcalfe C Rossotti N. Expanding agoraphobics’ horizons: An investigation of the effectiveness of A personal construct psychotherapy intervention. J Constr Psychol. (2006) 19:1–29. doi: 10.1080/10720530500311141

[80] Winter D Malighetti C Cipolletta S Ahmed S Benson B Röhricht F. Construing and body dissatisfaction in chronic depression: A study of body psychotherapy. Psychiatry Res. (2018) 270:845–51. doi: 10.1016/j.psychres.2018.10.061 30551334

[81] Bosworth HB Hays JC George LK Steffens DC. Psychosocial and clinical predictors of unipolar depression outcome in older adults. Int J Geriatr Psychiatry. (2002) 17:238–46. doi: 10.1002/gps.590 11921152

[82] Corrigan PW Phelan SM. Social support and recovery in people with serious mental illnesses. Community Ment Health J. (2004) 40:513–23. doi: 10.1007/s10597-004-6125-5 15672690

[83] Holvast F Burger H de Waal MMW van Marwijk HWJ Comijs HC Verhaak PFM. Loneliness is associated with poor prognosis in late-life depression: Longitudinal analysis of the Netherlands study of depression in older persons. J Affect Disord. (2015) 185:1–7. doi: 10.1016/j.jad.2015.06.036 26142687

[84] Leskelä U Rytsälä H Komulainen E Melartin T Sokero P Lestelä-Mielonen P. The influence of adversity and perceived social support on the outcome of major depressive disorder in subjects with different levels of depressive symptoms. Psychol Med. (2006) 36:779–88. doi: 10.1017/S0033291706007276 16566849

[85] Gonzales R Hernandez M Douglas SB Yu CH. Exploring the factor structure of a recovery assessment measure among substance-abusing youth. J Psychoactive Drugs. (2015) 47:187–96. doi: 10.1080/02791072.2015.1053556 PMC4732567 26134600

[86] Ostler K Thompson C Kinmonth A-LK Peveler RC Stevens L Stevens A. Influence of socio-economic deprivation on the prevalence and outcome of depression in primary care: The Hampshire Depression Project. Br J Psychiatry. (2001) 178:12–7. doi: 10.1192/bjp.178.1.12 11136204

[87] Barrera TL Cully JA Amspoker AB Wilson NL Kraus-Schuman C Wagener PD. Cognitive-behavioral therapy for late-life anxiety: Similarities and differences between Veteran and community participants. J Anxiety Disord. (2015) 33:72–80. doi: 10.1016/j.janxdis.2015.04.005 26005839 PMC4479977

[88] Schmidt LM Hesse M Lykke J. The impact of substance use disorders on the course of schizophrenia–a 15-year follow-up study: dual diagnosis over 15 years. Schizophr Res. (2011) 130:228–33. doi: 10.1016/j.schres.2011.04.011 21592731

[89] Trevillion K Shallcross R Ryan E Heslin M Pickles A Byford S. Protocol for a quasi-experimental study of the effectiveness and cost-effectiveness of mother and baby units compared with general psychiatric inpatient wards and crisis resolution team services (The ESMI study) in the provision of care for women in the postpartum period. BMJ Open. (2019) 9:e025906. doi: 10.1136/bmjopen-2018-025906 PMC6475160 30904867

[90] Galante JR Humphreys R Molodynski A. Out-of-area placements in acute mental health care: the outcomes. Prog Neurol Psychiatr. (2019) 23:28–30. doi: 10.1002/pnp.528

[91] National Health Service England. NHS long term plan (2019). Available online at: https://www.england.nhs.uk/long-term-plan/ (Accessed 2024 Feb 16).

[92] Emerald Publishing. Use a repertory grid (2023). Available online at: https://www.emerald (Accessed 2024 Mar 12).

[93] Hotopf M. The assessment of mental capacity. Clin Med. (2005) 5:580–4. doi: 10.7861/clinmedicine.5-6-580 PMC4953139 16411355

[94] Tawa J Suyemoto KL. The influence of race and power on self-construal in bicultural Asian Americans. Asian Am J Psychol. (2010) 1:275–89. doi: 10.1037/a0021388

[95] Tomico O Karapanos E Levy PD Mizutani N Yamanaka T. The Repeptory Grid Technique as a method for the study of cultural differences. Int J Des. (2009) 3:55–63.

